# Biolarviciding implementation in southern Tanzania: Scalability opportunities and challenges

**DOI:** 10.1371/journal.pone.0273490

**Published:** 2022-08-26

**Authors:** Athuman Yusuph Matindo, Eugene Benjamin Meshi, Ntuli Angyelile Kapologwe, James Tumaini Kengia, Stella Kajange, Prosper Chaki, David Zadock Munisi

**Affiliations:** 1 Department of Public Health, School of Nursing and Public Health, University of Dodoma, Dodoma, Tanzania; 2 President’s Office Regional Administration and Local Government, Dodoma, Tanzania; 3 Department of Environmental Health and Ecological Sciences, Ifakara Health Institute, Dar es Salaam, Tanzania; 4 Department of Microbiology and Parasitology, School of Medicine and Dentistry, University of Dodoma, Dodoma, Tanzania; Public Library of Science, UNITED KINGDOM

## Abstract

**Background:**

The resistance to insecticides among malaria vectors poses a global challenge in the efforts towards malaria elimination. This calls for an addition of larval control methods such as biolarviciding. However, the implementation of biolarviciding in Tanzania has been very low. Therefore, this study explored factors affecting the implementation of biolarviciding in the councils of Southern Tanzania.

**Methods:**

A mixed method descriptive qualitative, cross-sectional study design was used to collect data from 32 community leaders through key informant interviews and 12 Vectors Control Coordinators through in-depth interviews and questionnaire interviews and document review of implementation reports in 12 councils. Data were analysed using ATLAS.ti version 8, where content analysis was performed and SPSS for the quantitative data.

**Results:**

The study found low implementation of biolarviciding intervention in 9 out of 12 (75%) surveyed councils. All Vector Control Coordinators reported a shortage of at least one type of resources: funds, trained personnel, transport, supply of biolarvicide, and equipment; low community involvement (50%) and low level of community participation 83.3% (10/12).

**Conclusion:**

This study highlights resource inadequacy and low community participation as main barriers to the implementation of biolarviciding. Availing adequate resources and strengthening community participation through involvement in all stages of implementation is crucial for successful and sustainable implementation.

## Introduction

Malaria continues to be a disease of great public health importance in about 87 countries worldwide, including Tanzania [1]. In 2020, the disease was estimated to cause 241,000,000 morbidities and 627,000 mortalities globally; with the African region bearing more than 96% of the overall disease burden, with Tanzania being one of the four countries that contributed 53% of all the global Malaria deaths in the year 2020 [2].

The use of Long Lasting Insecticides Nets (LLINs) and Indoor Residual Spray (IRS) proved effective in combating Malaria in most African countries [3–5]. While this intervention continues to be a mainstay of malaria control. Yet, recent evaluation studies in different parts of the world suggests that these methods have no significant contribution to further the reduction of malaria prevalence [6, 7]. This could have been contributed by not only the emergence of resistance to insecticides (pyrethroids, organochlorines, and carbamates) among malaria mosquito species which have been reported in 68 countries [8], but also the unpredictability of community attitudes towards these methods [8–11]. Furthermore, a shift in the mosquitoes species from a predominantly indoor biting *Anopheles gambiae* to outdoor biting *Anopheles arabiensis* has also been proposed to be a limitation of the conventional mosquitoes control methods that have been in use for many years [3, 12]. These necessitated the World Health Organization (WHO)-Roll Back Malaria and Malaria Eradication Research Agenda for Consultative Vector Control group to emphasize the need to search for innovative complementary strategies such as larviciding, in order to achieve malaria elimination by 2030 [12, 13].

Larviciding refers to the “regular application of chemical or microbial insecticides to water bodies or water containers to kill the immature aquatic forms of the mosquito (the larvae and pupae)” [6]. Larviciding using microbial insecticides, also called ‘biolarviciding,’ effectively reduces vector density by indiscriminately controlling behaviorally plastic and insecticide-resistant vectors, safe and has no adverse impact on the environment [14]. Additionally, biolarviciding controls both malaria and non-malaria transmitting mosquitoes, such as *Culex spp* and *Aedes spp*, which can be helpful in the control of other mosquito-borne diseases, as such resources which could have been used in controlling non-malaria infections can be directed to the control of malaria vectors making it a promising tool to bring about malaria elimination [7, 15–17]. According to the national guideline on biolarviciding implementation, the implementation should involve the following steps: breeding sites identification, application of four rounds of biolarvicide a month, and surveillance to evaluate the presence of larvae after biolarvicide application to evaluate the effectiveness of the biolarvicide application [18].

In 2017, the Government of Tanzania scaled up nationwide biolarviciding in urban and rural areas to supplement the existing vector control interventions as a strategy for eliminating Malaria by 2030 [19, 20]. The government has established a factory for the production of Bacillus SH- strain 26612 (Bti) and *Bacillus sphericus* strain 2362 (Bs) to ensure its constant supply and availability domestically and beyond [21, 22]. To enhance the implementation of biolarviciding, the government distributed 236,420 litres of both types of biolarvicides (Bti and Bs) in all regions of mainland Tanzania for application in identified mosquito breeding sites. For biolarviciding to have an impact on reducing mosquito abundance and malaria prevalence, application coverage of not less than 50%, optimally above 70%, of all identified breeding sites is imperative [23–25]. However, environmental, logistical and financial challenges have been reported to hamper the successful implementation of microbial larviciding elsewhere [26, 27]. In the case of Tanzania, anecdotal reports indicated that, implementation coverage is low and inconsistent in some regions [20]. A report from a survey done by the Controller and Auditor General at the end of 2017 indicated that only 28% of distributed biolarvicide was applied among sampled councils [19, 20]. Similarly, a report from the survey by the President’s Office Regional Administration and Local Government (PO-RALG) [20] indicated that some councils had not completed the first-round application until the end of the year 2018. Multiple factors may affect implementation effectiveness, thus it is recommended to closely assess the progress of newly scaled vector control interventions brought into the community to assess the capacity, strengths, and weaknesses associated with the health systems [7]. This study was conducted to explore factors that affect the implementation of biolarviciding in Southern Tanzania to determine the opportunities and challenges associated with the scaling up of biolarviciding in different settings in Tanzania.

## Material and methods

### Study site

The study was conducted in the councils of Lindi and Mtwara, the regions in the Southern Zone of Tanzania. These regions have a high prevalence of malaria as indicated by the malaria indicator survey (2017), where the prevalence of malaria in under-five age children was 12% for Lindi and 15% for Mtwara. The southern zone regions are within a geographical location that lies between 9^0^30′S, 36^0^13′E, and 38^0^30′E, 39^0^08′E [28]. According to 2012 population census, ’the population of the zone is estimated at 2,135,506 population with 864,652 in Lindi region and 1,270,854 in Mtwara region [29]. The regions experience tropical climatic conditions with some variations both within and across the regions, characterized by a temperature that ranges from 23°C in July to 27.7°C in December and relative humidity ranging from 68% in October to 88% in March [30, 31]. The rainfalls assume a unimodal pattern, moderate that ranges from 1000mm to 1250mm, and extend between November and May with peaks in January and March [32–34].

### Study design and approach

This study was a cross-sectional descriptive study employing both quantitative and qualitative methods. The study populations were Vector Control Coordinators (VCC), village/street chairpersons and members of village health committees.

### Sampling and sample size estimation

A total of 44 participants, conveniently sampled, were selected for interview. Out of the 44 study participants, 32 were community leaders, i.e., village chairmen and members of the village committee, and 12 were VCCs. All selected community leaders and village committee members participated in Key Informant Interviews. This made a sufficient sample size for interviews of qualitative case studies, as suggested by Silva and Marshall [35]. In addition, semi-structured questionnaire was administered to 12 VCCs, and only five of them participated in an in-depth Interview based on a saturation point.

### Data collection methods

#### Key informant interviews

This was conducted with people who know what is going on in the community to collect information from a wide range of people, including community leaders; village chairmen, and the village committee members. These interviews aimed at understanding the community leaders’ awareness on the implementation of biolarviciding and the likely barriers stemming from resource availability, community participation, involvement in the identification, biolarvicide application, and program management in their villages and streets through community organisation, the contribution of resources, identification of the breeding sites, biolarvicide application and program management in respective villages or streets. The inclusion of community leaders was based on their role in implementing biolarviciding as stipulated in the national biolarviciding implementation guideline [18]. According to the guideline, the community leaders should be involved in locating waterbodies following up during biolarvicide application and evaluating the exercise. Therefore, because of the level of engagement, they are more informed than ordinary people in the community.

#### In-depth interview

In this study, In-depth interviews were conducted with VCCs to solicit their deeper understanding of how the biolarviciding exercise was conducted, and their views on barriers and facilitators for biolarviciding stemming from resource availability, community participation in the implementation of biolarviciding.Out of the 12 VCCs, in-depth interviews were conducted with five (5) of them after reaching saturation. VCCs were selected owing to their role as coordinators of biolarviciding implementation.

#### Document review

Reports on biolarviciding implementation by 12 councils were reviewed to verify implementation using a document review tool. The review tool was designed to collect information on establishing whether biolarviciding was implemented or not, and to assess the availability of biolarviciding resources, including funds, trained personnel, transport, supply of biolarvicide, and equipment.

#### Administration of a questionnaire and determination of resource availability

A semi-structured questionnaire was administered to twelve (12) VCCs to collect information relating to the availability of resources for the implementation of biolarviciding and the level of community participation in the implementation of biolarviciding.

Using a pre-designed template, the availability of resources was determined by comparing the resources supplied or available against the demand or budgeted amount.

#### Determination of the level of biolarviciding implementation

The level of biolarviciding implementation was obtained by reviewing the coverage of four components of biolarviciding as described in standard guideline for biolarviciding [18], which include coverage for identification of breeding sites, application of biolarvicide, effort level; the frequency of application, and larval surveillance in respective areas. Coverage of breeding site identification was given by the number of villages/streets to which identification of waterbodies is likely to serve as breeding sites. The coverage of biolarvicide application was defined as the number of breeding sites applied with biolarvicide divided by the total number of breeding sites identified in the area. The minimum recommended rounds of application are four (4), each round had 1 mark, and therefore full coverage of biolarvicide application had 4 marks. The coverage of larval surveillance (post-application) was defined as the proportion of breeding sites applied with biolarvicide where larval surveillance was conducted. A coverage of less than 50% for each component was scored as 0, while coverage of 50% and above was scored as 1. Therefore, the whole implementation has a total score of 6. The level of implementation was graded into low for scores 0 to 3 and high for a score greater than 3. The classification is based on implementation elements as stipulated in the guideline and other literature for larviciding [18, 23, 25].

#### The level of Community participation

The level of Community participation through VCC was assessed using the modified Spider gram model of participation described by [36] by focusing on four areas: involvement in need assessment, organization, resource mobilization, and program management; each carries a total score of 1. Involvement in need assessment was assessed through two options question asking whether the community was involved during planning for resources of biolarviciding (No = 0 Yes = 1). Organisation assessed the existence of group of people assigned a specific task and coordination among them (No task group = 0, existence of a group for a given task = 1/2, coordination = 1/2). When the score is 0 = No coordination; when no group assigned a specific task, 0.5 = Partial coordination; when there is an existence of a group of people assigned a specific task with poor coordination, and 1 = well coordination; when there is the existence of a group of people assigned a specific task with good coordination. Resource mobilisation assessed the leader’s assistance in availing resources for activity (No = 0 Yes = 1). Involved in management focused on community involvement in monitoring (0 = No 1 = Yes). The total score was 0 to 4. The level of community participation was then graded according to the model with minor modification into low for scores 0–2, and high for score of 3–4. Since there are 12 VCCs, the overall level of community participation would be regarded as low for total scores from 0 to 24 and high for scores from 25 to 48.

### Data collection tools

The interview guide, notebook, and audio recorder were used to capture information during the interview. The interview guide was prepared in English and translated into Kiswahili for convenience. The tool was adopted (modified) from the recommended tool for conducting scaling up case studies developed by WHO in collaboration with Expand Net and Management System International staff [37] and used in the study done by Quintero *et al*. [38].

### Data processing and analysis

Qualitative data from interviews were transcribed by the researcher and then translated into English by a Linguist. The transcribed and translated information was then entered into ‘ATLAS ti’ version 8 for data management and analysis. Content analysis of the transcribed information was done to provide groundedness (frequency of code responses) for each category of information. Data from the document review and Questionnaire interview was entered into SPSS version 20 for descriptive analysis, where by frequency and percentages were determined.

### Ethics statement

Ethical clearance for study and publication was sought from the University of Dodoma Institutional Research Review Committee. Informed verbal consent was sought from study participants before participation.

## Results

### Socio-demographic characteristics of study participants

A total of 32 community leaders were recruited for the study. The age of the study respondents ranged from 30 years to 69 years with a mean age of 51 years, with a median age of 52.5 years. The majority of interviewees were males 24 (75%), with most of them being within 25 to 54 years, age group 19(59.4%), had primary education 21(65.6%) and were peasants 17(53.1%) (Table 1).

**Table 1:** Socio-demographic characteristics of key informants (n = 32).

Variable	Frequency (n)	Percentages (%)
**Age group**		
25 to 54	19	59.4
55 to 64	10	31.2
Above 65	3	9.4
**Gender**		
Male	24	75.0
Female	8	25.0
**Education level**		
Primary	21	65.6
Secondary and above	11	34.4
**Occupation**		
Peasant	17	53.1
Businessman/Women	7	21.9
Others	8	25.0

A total of 12 VCCs were recruited for the study. Most were males 9(75%) and had a college-level education (75%). Among the VCCs, 6(50%) were clinicians, 5(41.7%) health coordinators and 1(8.3%) nurse. The majority 9(75%) of them had not received full training while only 3(25)% received full training on biolarviciding (Table 2).

**Table 2:** Socio-demographic characteristics of vector control coordinators (n = 12).

Variable	Frequency (n)	Percentages (%)
**Gender**		
Male	9	75.0
Female	3	25.0
**Education level**		
College	9	75.0
University	3	25.0
**Profession**		
Nurses	1	8.3
Health coordinators	5	41.7
Clinicians	6	50.0
**Biolarviciding training**		
Not trained	9	75.0
Trained	3	25.0

### Level of biolarviciding implementation

The coverage level of breeding site identification was 63.9%, while coverage of biolarvicide application was 73.8%, 34.7%, 15.3%, and 11.3% in the first, second, third, and fourth rounds respectively. None of the surveyed councils conducted larval surveillance. Out of 12 surveyed councils, 9(75%) had a low level of biolarviciding implementation coverage, while only 3(25%) had a high level of implementation coverage. Overall, the level of biolarviciding implementation in the study area was low (score of 2) (Table 3).

**Table 3:** Level of biolarviciding implementation (N = 12).

Council	a	b		C	d		e		f		g		h		j	k
	n	n%	Score	N	n (%)	Score	n (%)	Score	n (%)	Score	n (%)	Score	n (%)	Score		
Council 1	141	78(55.31)	1	296	296(100%)	1	80(27.03)	0	18(6.08)	0	12(4.05)	0	0 (0.00)	0	2	Low
Council 2	117	115(98.29)	1	385	315(81.81)	1	239(62.08)	1	200(51.95)	1	180(46.75)	0	0 (0.00)	0	4	High
Council 3	174	78(44.82)	0	300	300 (100.00)	1	0 (0.00)	0	0 (0.00)	0	0 (0.00)	0	0 (0.00)	0	1	Low
Council 4	158	66(41.77)	0	327	318(97.24)	1	0 (0.00)	0	0 (0.00)	0	0 (0.00)	0	0 (0.00)	0	1	Low
Council 5	127	112(48.82)	0	34	34(100.00)	1	0 (0.00)	0	0 (0.00)	0	0 (0.00)	0	0 (0.00)	0	1	Low
Council 6	110	70(27.27)	0	64	54(84.80)	1	32(50.00)	1	0 (0.00)	0	0 (0.00)	0	0 (0.00)	0	1	Low
Council 7	166	166(100.00)	1	360	360(100.00)	1	295(81.94)	1	190(52.70)	1	106(29.44)	0	0 (0.00)	0	4	High
Council 8	15	8(53.33)	1	8	8(100.00)	1	8(100.00)	1	8(100.00)	1	8(100.00)	1	0 (0.00)	0	5	High
Council 9	96	80(83.33)	1	15	6(40.00)	0	0 (0.00)	0	0 (0.00)	0	0 (0.00)	0	0 (0.00)	0	1	Low
Council 10	58	0(0.00)	0	0	0(0.00)	0	0 (0.00)	0	0 (0.00)	0	0 (0.00)	0	0 (0.00)	0	0	Low
Council 11	58	58(100.00)	1	856	327(38.20)	0	314(36.68)	0	11(1.28)	0	10(17.64)	0	0 (0.00)	0	1	Low
Council 12	152	45(29.60)	0	142	38(26.76)	0	0 (0.00)	0	0 (0.00)	0	0 (0.00)	0	0 (0.00)	0	0	Low
Overall	1,372	876(63.85)	1	2,787	2056(73.77)	1	968(34.75)	0	427(15.32)	0	316(11.34)	0	0 (0.00)	0	2	Low

a: Total number of villages/streets; b: Number of villages where breeding sites identification was conducted (Breeding sites identification coverage), n(%).

c: Number of breeding sites identified, n; d: Breeding sites with one round of application, n(%)*; e: Breeding sites with two round of application, n(%)*.

f: Breeding sites with three rounds of application, n(%)*; g: Breeding sites with four rounds of application, n(%)*; h: Breeding sites where larval surveillance was conducted n(%*); i: Total score; j: Level of implementation.

### Availability of resources for the implementation of biolarviciding

VCCs were asked about the availability and adequacy of resources for biolarviciding. All respondents reported a shortage of at least one type of equipment and absence of trained Community Own Resource Persons (CORPS), 9(75.0%) reported absence of training for VCCs, 7(58.3%) reported a lack of reliable transport, and 11(91.7%) reported a shortage of biolarvicide supply. Regarding fund availability, 10(83.3%) reported the council to have allocated funds, and 2(16.7%) reported that council endorsed adequate funds and in timely manner. Overall, all interviewees reported inadequacy of at least one type of resources for biolarviciding (Table 4).

**Table 4:** Availability of resources for implementation of biolarviciding (n = 12).

Variable	Availability (>50% of demand)	
	Adequate (%)	Inadequate (%)
**Transport**	5(42)	7(58)
**Biolarvicide supply**	8(67)	4(33)
**Skilled personnel**	0(0)	12(100)
Trained CORPS*	3(25)	9(75)
Trained Vector coordinators	2(17)	10(83)
**Equipment**		
Sketch map	3(25)	9(75)
Gumboots	3(25)	9(75)
Overalls	9(75)	3(25)
Dippers	2(17)	10(83)
Sprayer	11(92)	1(8)
**Fund availability**		
Allocation	2(17)	10(83)
Timely endorsed	2(17)	10(83)

* Community-Owned Resource Persons.

### Vector Control Coordinators’ perceptions towards community participation in the implementation of biolarviciding

We asked the VCCs about the extent to which they engaged community members in biolarviciding implementation using the Spider gram model as described by Chilaka [18]. Of the 12 respondents, 10(83%) suggested a low level of community participation, while only 2(17%) reported a high level of community participation. Further item analysis showed that about 6(50%) of the VCCs had involved community leaders in the needs assessment for biolarviciding implementation. However, more than half of the respondents 7(58%) reported poor organization among staff involved in biolarviciding; only 2(17%) reported to have been assisted by community leaders in mobilizing community members to volunteer for activities and equipment donation. Likewise, only 1(8%) VCC reported the involvement of community leaders in the biolarviciding program management (Table 5).

**Table 5:** Vector Control Coordinators’ perception on the level of community participation in the implementation of biolarviciding.

Variable	No, n (%)	Yes, n (%)
Involvement in need assessment	6(50.0)	6(50.0)
**Organization**		
No		
Partial		7(58.33)
Well		4(33.33)
**Resource mobilization**		1(8.33)
Equipment		
CORPS	10(83.33)	2(16.67)
**Program management**	10(83.33)	2(16.67)
	11(91.67)	1(8.33)

### Community views on biolarviciding implementation

Table 6 indicates the key informants’ responses to various elements about biolarviciding implementation. Out of thirty-two (32) interviewees, 17(53.1%) interviewees were aware of Biolarviciding implementation in their areas. Of these, 10(31.3%) reported having received advocacy in their community meeting while others reported receiving information from other sources or when they witnessed the biolarvicide application. It was found that, 15(46.9%) of the respondents, reported biolarvicide application in their areas any time before, while only 7(21.9%) reported the application for the year 2019.

**Table 6:** Content analysis for key informants’ views on the implementation of biolarviciding (n = 32).

Variable	Category	Frequency(%)
**Awareness on biolarviciding**	Aware	17(53.1)
	Unaware	15(46.9)
**Advocacy on biolarviciding**	Advocacy	10(31.3)
	No advocacy	22(68.8)
**Conduction of breeding sites identification**	Conducted	15(46.9)
	Not conducted	17(53.1)
**Biolarvicide application**	Applied 2019	7(21.9)
	Not applied 2019	25(78.1)
	Applied anytime	15(46.9)
	Never applied	17(53.1)

*I ever heard of that. There were gallons of watery chemicals brought to our office from the health department for spraying in toilets (KI 22, Male, Community leader)*.

*When they came here, they approached me. They planned to apply biolarvicide the whole Ward, every street. First, they started in my area, then went to Ufukoni Street (KI 7, Female, Community leader)*.

Some interviewee responses suggested inconsistent application of biolarvicide in their areas. One interviewee reported that it had been a long time since the application of biolarvicide was conducted around their environment.

*They did it once, and they did not come back again. I don’t know! Maybe the Government does not have enough chemicals, or it is the ineffectiveness of the people employed to apply biolarvicide. I don’t know. But there is a very infrequent application of biolarvicide here (KI 7, Female, Community leader)*.

Among those who were aware of biolarvicide intervention, some had heard about but never seen its implementation in other areas of the council. A total of 17(53.1%) reported not having seen the application implemented or been involved in identifying mosquito breeding sites. One respondent shared that:

*No [breeding site identification] Not at all*. *But there are many places with standing water*. *For example*, *we have that canal that accumulates a lot of trashes from town*. *When it rains*, *all kinds of trashes are brought here by the flood*. *So this place becomes a host of mosquitoes and trashes* (KI 5, Female, Community leader).

Some informants reported the matter to have been discussed at the community meeting though it had not come into effect. When asked to explain their understanding on biolarviciding, one interviewee responded that:

*We had a meeting last month and the chairman introduced that issue; he noted that there would be this activity in the near future though he didn’t give details on how it will be done* (KI 21, Male, Community leader).

Another informant reported that the issue had been planned a long time ago but had never been implemented. When asked whether biolarviciding was implemented or even discussed in any community meeting, he responded that:

*It could be 2018 or 2019*. *It was just tabled and discussed in the ward development committee meeting*. *After that discussion*, *I have never seen its implementation*. *I have never seen the officials coming to our street to say ‘now we have come to biolarvicide’* (KI 2, Male, Community leader).

Fifteen (46.88%) of interviewees who had ever heard about biolarviciding were not aware whether the implementation took place in their areas while 5 (15.6%) had never heard of biolarviciding at all:

*If that [biolarviciding] is happening*, *maybe it is done for an individual family or person*, *but not something communal*. *I know there was spraying of chemicals in that pond in previous years but not in recent years* (KI 20, Male, Community leader).

Other respondents opined that there was a lack of involvement of political leaders who could assist in sensitizing the community on biolarviciding. He stated that

*I was going to say that; political leaders have not been open about this*. *The community [members] has not been put open about this* (KI 15, Male, Community leader).

In some areas, interviewees stated that they had several meetings with health officials regularly providing education on malaria prevention. yet there had never been community sensitization on biolarviciding. At the same time another highlighted that the official had not undertaken enough community sensitization, and the task of providing education to the community members had been left to community leaders despite their limited education regarding biolarviciding.

*I can say that the officials in meetings just informed us about the effects of Malaria*. *It is a very difficult exercise to fight against Malaria*, *it needs collaborative efforts to fight against mosquitoes*. *I have not seen the officials educating people about biolarviciding in mosquito breeding sites* (KI 15, Male, Community leader).

*First of all*, *the community would like to do it in a way that considers their safety… the Health Department only depending on sub-village leaders to educate people without getting down to collaborate with the community leaders to educate them would not be effective*. *If possible*, *the community should hear what health coordinators say* (KI 17, Male, Community leader).

### VCC views on the implementation of biolarviciding

When asked whether the implementation was done or not and to explain on how it was implemented in their respective councils, all interviewees affirmed biolarviciding being implemented in their councils on different scales. They explained when it began and the approach adopted.

*This activity started in our council in 2018*,…*biolarvicide was brought to our council and we started making awareness campaigns around the community through the community leaders*. *Thereafter*, *we identified the breeding sites… Then*, *having identified the area*, *we started larviciding according to the ways we planned* (IDI 3, Female, VCC).

Another interviewee suggested the implementation was done in the year 2019 and had planned to continue with the implementation even for the year 2020.

*We are implementing biolarviciding*, *and last year the government selected regions with a high burden of Malaria*, *and our Region was among them*. *So we received biolarvicide from national allocation…In September [2019]*, *we applied to the breeding sites…we are expecting to spray in the coming July [2020]* (IDI 4, Female, VCC).

Interviewees claimed that there was inconsistent and infrequent application due to some challenges.

*To my knowledge*, *we were instructed to apply once weekly*, *had to keep applying until there were no larvae in a particular breeding site; however*, *we don’t get to that stage*. *We cannot afford the running cost* (IDI 4, Female, VCC).

Other interviewees pointed out that biolarvicide application was not conducted in some areas because they had no breeding sites.

*We managed to spray in areas where there were breeding sites*, *though in different years*. *Some Wards don’t have breeding sites* (IDI 4, Female, VCC).

Interviewees reported that they do not conduct larval surveillance to confirm larvae presence in water bodies suspected as breeding sites for mosquitoes. Similarly, there is no larval surveillance to test the effectiveness after application:

*Frankly speaking*, *the exercise is not done in accordance with the established standards*. *This is because the standards require one to have estimated the number of larvae in each breeding site before application [which was not done]* (IDI 1, Male, VCC).

When asked why some community members are not aware of the biolarviciding? One interviewee described the challenge in gathering community members while another confessed to have not conducted advocacy to community leaders due to financial challenges:

*This is because we sit with them in ward meetings and street meetings*, *and we tell them something will be done and it will be done on a particular day and time*. *Many citizens don’t attend meetings when we call; you may find only ten people attending a meeting…We talk to the ten people because we can’t easily get 30 or 40 people to attend meetings here*. *We tell those who attend meetings to inform their neighbours that the exercise will be done on a specific date* (IDI 3, Male, VCC).

*(…) we met with Ward Executive Coordinators*. *However*, *in accordance with the guideline we were supposed to meet with councilors*, *and street chairmen; so that they get informed*, *so it is easier for them to sensitize the community members*, *but we didn’t manage because it needed much money* (IDI 4, Female, VCC).

Another respondent explained that most of the time, officials share the information regarding various health interventions with the community leaders expecting that the leaders will share the same with the community members during community meetings. When this information is not shared the community remains unaware of the intervention:

*It is possible we went to apply before the message was delivered to them*. *Leaders would share information like this in meetings*, *but the activities begin before the meetings*, *so the community remains unaware* (IDI 1, Male, VCC).

On the other hand, the interviewees attributed the lack of awareness of the community members to biolarviciding to it being conducted in isolated places, out of sight of community members:

*You know*, *it is not always that they know what is going on because we do not do the activity in open spaces but only in breeding areas*, *and these areas often depend on*: *terraces*, *standing water and*, *in other ward*, *there is no standing water at all* (IDI 2, Male, VCC).

Others accepted that some community members might remain unaware since the activity was not done in all Wards.

*We could not reach all wards [for biolarviciding]* (IDI 3, Female, VCC).

Regarding coverage of breeding site identification and biolarviciding, interviewees reported difficulty reaching all breeding sites since some of the breeding sites are inaccessible areas.

*Accessing all these areas is a challenge*. *Technically*, *it is said that for you to be able to destroy all the breeding sites; they must be fixed*, *few*, *and findable*. *However*, *in this District*, *few breeding sites fulfil these criteria*. *Others are not reachable because they are in remote areas where not reachable* (IDI 1, Male, VCC).

### Community leaders’ views on the availability of resources

Some interviewees who engaged in the activity reported inadequacy of biolarvicide as one of the barriers to effective implementation of biolarviciding. One interviewee was quoted as saying that “*They informed us [about biolarviciding] and*, *fortunately*, *they provided the biolarvicide*. *They were not enough for all the areas we have here*. *So*, *we did it in phases”* (KI 17, Male, Community leader).

### VCC perceptions on the availability of resources for biolarviciding

Interviewees reported inadequate resources including equipment, trained personnel, the supply of biolarvicide and transport facilities. The lack of funds was identified as the main barrier to implementation. One interviewee reported a lack of adequate preparation for biolarviciding implementation as the main cause, since directives to begin the implementation of biolarviciding including the ministry of health sending biolarvicide in councils was effected without prior council plan for activity, including allocating funds for biolarviciding:

*I can say that the exercise was ineffective because the activity was abruptly brought to use*. *They just brought the larvicides here*. *There was no significant preparation… You know*, *if you want to assign people to an activity*, *there must be a budget; there was no budget at all* (IDI 3, Female, VCC).

Despite the fact that most interviewees reported that the councils have allocated some money for biolarviciding in their budget, most of them reported that the fund was inadequate. When asked about the underlying cause, some of the interviewees’ responses suggested low council financial capacity

*Based on the financial capacity of the council*, *we cannot reach all*. *Had there been NGO or donor that supports it*, *we can manage it because we don’t have many breeding sites* (IDI 5, Male, VCC).

Similarly, another interviewee noted that while the council tried to ensure the availability of funds through the health facilities budget, resources remained insufficient.

*We have severally asked for funds from the council for this activity*. *The council heard us and set some budgets in some service centres to support the activity though it was not enough* (IDI 1, Male, VCC).

Another interviewee attributed the financial insufficiency to a lack of commitment among the council management.

*I think the councils cannot fail to provide that amount of money*. *They need to get an order from their superiors*, *because I can remember what happened past two years*, *there were directives to directors*, *and they made follow-ups on the implementation of biolarviciding*. *Before that biolarvicide would be brought and end up being stocked at the store*, *but after that directive*, *we could see them emphasising the implementation* (IDI 4, Female, VCC).

Lack of commitment was perceived as one of the barriers by another interviewee as he noted that there is no interest among the superiors in the council regarding the implementation of biolarviciding.

*You see*, *we people often want to see someone standing for something so we can stand on the right path…had there been someone[superior] advocating for biolarviciding and making a follow-up*, *we would have gone far*. *The leaders from different levels had given it attention; we could succeed* (IDI 2, Male, VCC).

Interviewees reported insufficient biolarvicide supply. While there was an understanding of the amount required for application, a supply shortage prevented them from implementing according to a standard guideline.

*(…) we have some problems*. *According to the guideline*, *if you read; spraying need to be done every week for at least three months*. *But its implementation is challenged by a lack of funds and biolarvicide supply*. *We often receive biolarvicide from the government*. *For example*, *last year we received 1400 litres*, *the amount that was sufficient for only six ponds [out of eight]*, *and we applied only one round* (IDI 5, Male, VCC).

When probed why there were biolarvicide shortages supplied by Ministry of Health, interviewees reported lack of involvement of officials at lower levels in the quantification process.

*I don’t know what their base for this estimation was*. *Last year they ordered to identify breeding sites and provide an estimation for the required amount of biolarvicide*. *When we sent it to them*, *they gave us 1400 litres*. *However*, *our estimation was 3000 litres*. *And because it is given free*, *you just accept* (IDI 5, Male, VCC).

One respondent suggested that we can reduce cost by ensuring the presence of local agents supplying biolarvicide. She said that;

*Imagine travelling to Kibaha*, *and you end up buying only 5 gallons that become expensive*. *But if it is locally available*, *it becomes cheaper* (IDI 4, Female, VCC).

Another challenge described by interviewees was inadequately trained personnel due to failure to train dedicated staff for biolarviciding; VCCs, and Community Own Resource Person. Consequently, the coordinators were forced to use untrained personnel such as Community Changing Agents (CCA); community members who volunteer for various health activities in the community.

*I mostly relied on CCA who received training*, *but it was insufficient to make them competent enough to identify larvae*. *It was one-day training just to brief them* (IDI 4, Female, VCC).

Lack of training hindered the involvement of community members ready to volunteer for activity.

*I have to be honest; there has been no training for the people involved in the biolarviciding activity*. *There was also no representation of residents from each street or ward* (IDI 1, Male, VCC).

Alternatively, some relied on health coordinators believing they are competent in the area since larviciding is one of the topics they learn during their training.

*We just use health coordinators*, *since one of the topics they had during their studies was biolarviciding* (IDI 5, Male, VCC).

One interviewee reported that, because of lack of equipment, they don’t test for the presence of larva in water bodies before application or undertake larval surveillance post application.

*We don’t have that equipment*. *We just examine it through our eyes*. *We walk around the water bodies to look for mosquitoes*, *area with mosquitoes are assumed to have larvae* (IDI 36, Male, VCC).

When asked why no larvae testing was done after, interviewees reported having no adequate skills and lack of equipment for performing this test.

*Because of the shortage of facilities*, *we used the following ways*. *We could identify through our own eyes all the places that have clean standing water*, *[since] clean standing water contains larvae*. *We used these criteria* (IDI 1, Male, VCC).

Another interviewee gave a similar view, who believes that all clean standing water contains larvae.

*We mean*, *the areas with standing water must have larvae; we applied biolarvicide in all the areas with standing water*, *be it a permanent or temporary breeding site* (IDI 3, Female, VCC).

Transport was another challenge pointed out by some interviewees. It was reported that some councils have no special car for malaria activities and therefore depend on ambulances. This hinders the frequent application of the intervention in distant areas.

*As I said earlier*, *this activity needs to be done daily [regularly]; see*, *we use the ambulance to take people going for activity*, *but also*, *we put the larvicides in it; it has a strong smell*. *We need a vehicle that withstands driving on roads that are difficult to pass* (IDI 9, Male, VCC).

### Community leaders’ views on community participation in biolarviciding

The interviewees played different roles in facilitating the implementation of biolarviciding, from identifying of breeding sites to its application. One interviewee described their participation in breeding site identification.

*As a chairperson*, *I have to show them all the areas with standing water*. *So I showed them all water that runs from toilets and all areas with standing water* (KI 7, Female, Community leader).

Another interviewee described his participation in biolarvicide application. *“I have participated as the implementer of the activity”* (KI 3, Male, Community leader). However other interviewees reported limited involvement in biolarviciding. They reported having been involved only during planning, while a few reported being involved during the implementation while the remainder were not involved at any stage.

*We have never been consulted but heard that some people move around our areas to apply biolarvicide in those areas with standing water*. *However*, *we do not know anything about it because they never told us they would come to our area on a specific day* (KI 2, Male, Community member).

Another interviewee showed not to be aware of the implementation but suggested that it was important to involve the community for program sustainability.

*If they applied without informing us*, *I beg them that in the next round*, *they should involve us so that we can understand what they do and sustain it* (KI 35, Male, Community member).

### VCC views on community participation in biolarviciding

The interviewee affirmed that the community has been participating in the activity when involved.

*They show to be understanding*. *We consulted sub-village leaders*, *and they helped us find the youths to work with*, *and in the end*, *the activity was done*. *[That means] people understand it*, *they agreed to work with us* (IDI 3, Female, VCC).

*The community members collaborate by showing us the areas with mosquito breeding that we do not know about*. *They tell us when there is a place we forget* (IDI 2, Male, VCC).

Another interviewee described the importance of involving the community in that, besides providing support, it builds trust with the community members of the programme.

*In each ward*, *a person involved in applying biolarvicide*. *This makes them an advocate for the exercise to the community members* (IDI 5, Male, VCC).

One interviewee shared the approach they use to involve the community: sending a message through a letter and advocacy in community meetings.

*First*, *we inform them of the exercise through a letter*. *But on the day of application*, *we go with him*, *because community members regard us as guests; they know their leaders* (IDI 2, Male, VCC).

When probed why some of the community leaders were not involved in the activity, interviewees reported that leaders are involved during meetings, while other reported that leaders are not always available in the meetings or during the activity. At the same time, it was apparent that community members and their leaders are not fully involved in the planning and implementation’ they are only informed of the activity.

*We normally attend full council meetings where ward leaders are members and representatives of village leaders*. *So*, *all directives that we want to reach to villages*, *we deliver to District Medical Coordinator*, *who also attends full council meetings*, *and we believe the messages reach the village leaders* (IDI 5, Male, VCC).

*Because of life challenges*, *the street leaders are not found in their areas when the Heath Coordinator reaches them; they are in different areas* (IDI 2, Male, VCC).

One interviewee reported that community members who participate in biolarviciding activity are paid rather than voluntarily.

*We pay them*. *As I told you*, *we requested funds for this activity*. *Unfortunately*, *the fund was not all released*. *So*, *for the amount endorsed*, *we hired a spraying machine and paid these laborers for the rest*.. *We paid them Tshs*. *30*,*000 each* (IDI 5, Male, VCC).

Another described that the community member came into the program as a volunteer, but they wanted to be paid in the long run.

*The group has been very helpful in facilitating this activity*. *However*, *they started as volunteers initially*, *but in the end*, *they started demanding for allowances and when the district council could not pay them*, *they support was decreased* (IDI 1, Male, VCC).

A similar contention was raised by another interviewee who reported having not paid the community members but managed to continue with the activity.

*They asked for an allowance*, *but we had no money to pay them*. *So you must motivate them*, *and they asked*, *but we had nothing to provide* (IDI 3, Female, VCC).

When probed why some community leaders were not involved, interviewees reported that sometimes not all leaders are accessible.

*I passed in some areas to inform them*, *but those in distant areas were not effectively involved informing the whereabouts of the activity* (IDI 9, Male, VCC).

The findings from key informants are consistent with the findings in the document review where it was observed that there was a low supply of biolarviciding and a shortage of resources.

## Discussion

In its efforts to achieve malaria control and elimination, in 2017, Tanzania scaled up nationwide biolarviciding against Malaria vectors [20]. This study was conducted to assess factors related to early reports suggesting ineffective implementation of biolarviciding among councils in Southern Tanzania. Our results reflect perceptions of both implementers and receiving communities as to the challenges and opportunities associated with scaling of biolarviciding in the region.

This study found an ineffective implementation of biolarviciding. This is similar to what was reported in the President’s Office, Regional Administration and Local Government (PO-RALG) [20] and Control and Audit General (CAG) [19] surveys that reported that, most of the councils did not implement one or more of the implementation components. Studies have shown that when microbial larviciding [biolarviciding] for malaria control is effectively implemented, it may significantly reduce the mosquito population and malaria burden [40]. An effective biolarviciding requires identification of almost all mosquito breeding sites, and at least 50% should be applied with a biolarvicide [1, 14]; however, this study found that the coverage level of breeding site identification was only 63.85%, and only the first round application managed to attain this minimum recommended application coverage (50%) while this coverage progressively decreased in each subsequent application rounds. Regular application is recommended to maximize the effectiveness of interventions such as biolarviciding [18, 39]. However, this study found that, biolarvicide application was conducted only once in most surveyed councils. This resulted from a lack of resources caused by failure to allocate sufficient funds for biolarviciding implementation. This inconsistency in the application could allow for re-establishment of mosquito breeding sites, reducing the intervention’ impact. Therefore, unless there is an improvement in coverage and frequency of biolarviciding, the expected benefits of the novel intervention in the country will not be realized. As a result, malaria will continue to proliferate, and community members may lose interest in participating in the implementation process, which is necessary for the sustainability and overall success of the Intervention.

Larval surveillance is another important component of biolarviciding implementation [14, 40]. All potential breeding sites need to be tested for the presence of larvae before and after application of biolarvicide [18]. This study found that larval surveillance was not conducted in all surveyed councils. Since not all water bodies have larvae, applying biolarviciding universally wastes energy and resources and increases costs associated with the intervention. Likewise, failure to conduct larval surveillance after application hinders the evaluation of the intervention; thus, the cost-effectiveness cannot be adequately estimated [41].

Success in biolarviciding implementation in councils of southern Tanzania is limited by resource inadequacy. The study revealed that most councils faced a shortage of at least one resource type. Most councils had inadequate skilled personnel for biolarviciding implementation. It was found that most of the staff, at council and ward levels had not received formal training on biolarviciding. This might result from inadequate budgeting for on-job training in the study area. As per Derua *et al*. [42], there is a need to build the capacity of staff involved in newly scaled interventions; however, our study has found that training was not undertaken as part of the implementation. Training ensures that involved staff have the requisite knowledge and skills for intervention implementation [18]. Consequently, important components were not implemented, particularly larval surveillance,.

Effective implementation of biolarviciding also requires availability of adequate and suitable equipmentat the time of the activity [14, 43]. In this study, it was found that councils lacked some of the equipment identified in the national guideline as important for larval surveillance (e.g., dippers, writing boards, sketch maps), biolarvicide application (knapsack sprayers), and personal protection (e.g., gumboots, overalls, plastic gloves) [18]. Under such circumstances, councils had to rely on hired or locally donated equipment. This resulted from ineffective planning and budgeting prior to the start of the biolarviciding implementation, which had two implications; delays to the implementation process and provided room to skip the relevant task, as was the case with larval surveillance. Another resource found inadequate was the supply of biolarvicide. Shortage of biolarvicide supply was reported by Vector Control Coordinators in one-third of surveyed councils. This contradicted the findings from the survey done by CAG [19], which found that only 28% of supplied biolarvicide was used. However, it was noted that the implementation of biolarviciding started late in 2018 and was initiated without councils having allocated resources to the activity. Thus, there was a lack of resources at the council level for preliminary activities such as advocacy meetings and sites identification. Biolarvicide distributed in early 2018 went unused until late 2018 when the implementation took off in most of the councils. There was inadequate coordination when it came to quantification, which was mainly done at a Ministerial level without full involvement of the Vector Control Coordinators. This caused an accumulation of biolarvicide at the council level and was falsely interpreted as an over-supply of biolarvicide related to demand. However, when implementation begun, the quantity delivered was short of the actual demand.

Fund insufficiency was identified as the main cause of resource inadequacy. Among the underlying causes was low commitment among council management in allocating and timely endorsing sufficient funds. Influencing factors to include lack of innovative domestic resource mobilization for health interventions which is partly contributed by less priority given to the health sector and donor dependency mind-set when it comes to malaria interventions [44–46]. However, Fillinger *et al*. [39], asserts that “the true programmatic value of larviciding though can only be established through longer-term programmes which are stably financed and allow the capacities of operational teams and infrastructures to mature through direct experience of locally relevant challenges”. It is therefore necessary to capacitate councils with necessary resources to gain momentum before leaving them full control on implementation process.

The implementation of biolarviciding in Tanzania adopts community-based approach, in which the community had to take part in activities such as breeding sites identification and application of biolarvicide [18]. However, this study showed some community members did not take part in the implementation. One of the underlying factors pointed out was lack of community involvement. Low community involvement has previously been reported by Waibe [47] in a study done in Iringa region, southern highlands of Tanzania. Another factor found in this study was lack of volunteerism among some community members, who wanted to be paid for the activities they took part. Since there was no money allocated for these community members, they decided not to continue participating in the implementation of biolarviciding.

Proponents of community participation urge that involving the community in intervention promotes community ownership and encourages them to contribute resources, engage in activities involved in the intervention, provide ideas during design and implementation and assist in management [48] which is vital for program sustainability. Therefore VCCs need to strengthen community involvement. Similarly, VCCs, in collaboration with the community leaders, need to fully educate the community on the program’s benefits for them to be willing to volunteer for the work.

We acknowledge that this study might have been limited in a number of ways. First of all, the study was based on community leaders’ self-reporting. Given the time lapse between implementation and the study, respondents could have forgotten some aspects of implementation. Similarly, interviewees’ responses could have been influenced by feeling they are well identified as leaders therefore introducing response bias.

## Conclusions and recommendations

This study has revealed opportunities and challenges facing the implementation of biolarviciding in Southern Tanzania. The main identified challenges facing the implementation were resource scarcity: inadequate funds, inadequately trained personnel, inconsistency in biolarvicide supply, inadequate equipment, and unreliable transport for carrying out biolarviciding activities. Another challenge was inadequate coordination between the councils and the Ministry of Health during the quantification of biolarvicide, resulting in frequent biolarvicide stock-outs. On the other hand, the study found some opportunities for biolarviciding implementation that manifested through the existence of vector control coordinators, the existence of a budget for biolarviciding in some of the councils that were set for the implementation of biolarviciding, the existence of local industry that produces biolarvicide and existence of community members who are willing to take part in the biolarviciding, a potential factor for program achievement and sustainability. Therefore, despite the identified challenges, implementing of biolarviciding in Southern Tanzania was feasible and potentially sustainable when identified challenges are resolved.

We recommend that the Ministry of Health, through National Malaria Control Programme in collaboration with the President’s Office Regional Administration and local government (PORALG) and implementing partners, conduct training on biolarviciding to all Vector Control Coordinators in Southern Tanzania and mobilize sufficient resources for the implementation of this intervention. On the other hand, councils need to allocate sufficient financial resources from their own sources and disburse them timely to ensure adequate material and equipment availability for biolarviciding. Adequate materials and skilled personnel will help ensure effective implementation of biolarviciding at a scale that will accelerate attainment of the control and elimination of malaria.

To evaluate the effectiveness of intervention and account for value for money invested in the intervention, Vector Control Coordinators need to ensure larval surveillance is conducted before and after the application of biolarvicide. Such information is vital for attracting stakeholders’ investment in the intervention.

To ensure program sustainability, we recommend that Vector Control Coordinators improve community involvement in all stages of implementation. This will create program ownership among community members and inspire community readiness to use their resources in the future.

## Supporting information

S1 Data Biolarviciding implementation in Tanzania dataset. (SAV)

S2 Data Biolarviciding implementation in Tanzania data collection tools. (DOCX)
