# Supplementary material for: Biolarviciding implementation in southern Tanzania: Scalability opportunities and challenges
Source: PLoS One. 2022 Aug 26;17(8):e0273490. doi: 10.1371/journal.pone.0273490 (PMC9417020; doi:10.1371/journal.pone.0273490)
Supplement: S2 Data — (DOCX) [file pone.0273490.s002.docx]

**Interview guide to Key informants__English version**

1. **Respondent particulars**

1.1 Number……..

1.2 Age………….

1.3 Gender….……

1. **Biolarviciding implementation**

2.1 What do you know about the implementation of biolarviciding in the /village?

2.2 Can you describe how you were involved in designing and assessment of needs for biolarviciding implementation?

2.3 How you and other member organised to carry out different tasks for biolarviciding? How are you engaged in program management and follow up to assess the result of the program? (If not involved what is the willingness?)

2.4 How you and other members of the community are participating in resource mobilisation for biolarviciding implementation? (If not involved what is the willingness?)

2.5 Were mosquito breeding sites identification done in the whole of this street/village? If not what was the reason for failure?

2.6 Were all identified breeding sites treated with biolarvicides? If not all identified breeding sites were applied with biolarvicide, what could have been the reason for not treating all the identified breeding sited?

2.7 Was there surveillance done after application?

2.8 How do the village leaders involved in the biolarviciding implementation?

2.9 Was there any community sensitization on biolarviciding implementation in your area? How do other political leaders were involved in community sensitization for biolarviciding?

1. **Facilitating factors**

3.1 In your opinion, what factors facilitated successful implementation of biolarviciding in your village?

3.2 In your opinion, what factors influenced community participation in the implementation of biolarviciding in your village?

3.3 What is the likelihood that your participation may be sustainable in future?

1. **Barriers for larviciding implementation**

4.1 In your opinion, what could have caused any failure in the implementation of biolarviciding in your village? What was done in an attempt to mitigate the problem? Was it successful? If not, why? Was there any other solution?

4.2 Is there a need for change or improvement in village or council management or legal aspects for successful participation in biolarviciding given the barriers identified?

**Interview guide to Key informants__Swahili version**

1. **Taarifa za mshiriki**

1.1 Namba: ………………..

1.2 Umri……………………

1.3 Jinsi……………………..

1.4 Kiwango cha elimu……………

1. **Utekelezaji wa upuliziaji wa viuadudu**

2.1 Wafahamu nini kuhusu zoezi la upuliziaji wa viuadudu katika kijiji chako?

2.2 Je! Waweza eleza ni kwa namna gani wanajamii mlishirikishwa katika kupanga namna ya kutekeleza na kutathmini mahitaji ya utekelezaji wa upuliziaji wa viuadudu?

2.3 Ni kwa namna gani wanajamii mmejipanga kwa utekelezaji wa zoezi hili? Ni kwa jinsi gani mmeshiriki kutekeleza ikiwa ni pamoja na kufuatilia maendeleo na matokeo yake?

2.4 Ni kwa namna gani wanajamii mlishiriki katika kuchangia rasilimali( fedha, nguvu na vifaa) kwa ajili ya upuliziaji wa viuadudu?

2.5 Je! Zoezi la kusaka mazalia ya mbu (maji ambayo yana viluwiluwi au uwezo wa kuwa chanzo cha mbu kuzaliana) lilifanyika katika maeneo yote ndani ya kijiji hiki?

2.6 Je! Mazalia yaliyo gundulika ni yote yaliweza kupuliziwa na dawa ya viuadudu? Kama sivyo ni sababu zipi zilipelekea kushindwa kupulizia viuadudu kwenye mazalia yaliyo salia?

2.7 Je! Ufuatiliaji ulifanyika kutathmini manufaa ya upuliziaji?

2.8 Je! Ni kwa namna gani viongozi wa kijiji mnashiriki katika zoezi la upuliziaji viuadudu?

2.9 Je kulikuwa na uhamasishaji juu ya zoezi la upuliziaji wa viuadudu katika jamii?ni kwa namna gani viongozi wa siasa wanashiriki katika uhamasishaji wa jamii juu ya upuliziaji wa viuadudu?

1. **Sababu wezeshi kwa utekelezaji wa upuliziaji viuadudu**

3.1 Ni sababu zipi zimekuwa chanzo cha mafanikio ya zoezi la upuliziaji kwa kiwango kilicho fikiwa?

3.2 Ni sababu zipi zimeweza kuchochea ushiriki wa wananchi katika zoezi la upuliziaji?

3.3 Je! Kuna uwezekano wa ushiriki wenu kuwa endelevu huko mbeleni?

1. **Sababu zuizi kwa utekelezaji wa upuliziaji viuadudu**

4.1 Nis sababu zipi zimepelekea ufanisi wa upuliziaji kuwa duni? Je! Ni mbinu zipi ziliwahi tumika kutatua changamoto hizo? Je! Zilikuwa na manufaa? Je! Kuna namna nyingine ya kutatua changamoto hizo?

4.2 Je! Kuna uhitaji wa kufanya mabadiliko au kuboresha namna ya usimamizi au sheria (hapa kijijini/mtaani au kwa halmashauri) ili kuongeza ushiriki wa wanajamii katika zoezi hili la upuliziaji wa viuadudu?

**Interview guide for Indepth Interview/Vector Control Officers**

1. **Respondent particulars**

1.1 Number……..

1.2 Age………….

1.3 Gender….……

1. **Biolarviciding implementation**

2.1 What do you know about the implementation of biolarviciding in your council?

2.2 What factors do you think influenced the decisions to adopt biolarviciding as malaria mosquito control method? How strongly do you see these factors were important and relevant to your settings?

2.3 Do you see biolarviciding implementable (*feasible*) and appropriate to your council?

2.4 Can you describe how the community were involved in biolarviciding implementation?

2.5 Can you explain how were the community leaders involved in the biolarviciding implementation?

2.6 Were mosquito breeding sites identification done in all streets/villages of this council? If not what was the reason for failure?

2.7 Were all identified breeding sites treated with biolarvicides? If not all identified breeding sites were applied with biolarvicide, what could have been the reason for not treating all the identified breeding sited?

2.8 Was there surveillance done after application?

2.9 What is your comment on the situation of resources availability for biolarviciding in your council? (Depending on availability of resources found in document review)

1. **Facilitating factors**

3.1 In your opinion, what factors facilitated successful implementation of biolarviciding in your village?

3.2 Is there evidence that the biolarviciding can be sustainable in this council?

3.3 In your opinion, what factors influenced community participation in the implementation of biolarviciding in your village?

3.4 What is the likelihood that your participation may be sustainable in future?

1. **Barriers for larviciding implementation**

4.1 In your opinion, what could have caused any failure in the implementation of biolarviciding in your village? What was done in an attempt to mitigate the problem? Was it successful? If not, why? Was there any other solution?

4.2 Is there a need for change or improvement in village or council management or legal aspects for successful participation in biolarviciding given the barriers identified?

**Interview guide for IDI__Swahili version**

1. **Taarifa za mshiriki**

1.1 Namba: ………………..

1.2 Umri……………………

1.3 Jinsi……………………..

1.4.Kiwango cha elimu……………

1. **Utekelezaji wa upuliziaji wa viuadudu**

2.1 Wafahamu nini kuhusu zoezi la upuliziaji wa viuadudu katika Halmashauri yako?

2.2 Wadhani ni sababu zipi zilipelekea uamuzi wa kuanza kutumia viuadudu kama njia ya kudhibiti mbu? Je wadhani sababu hizo zina mashiko na ni zina endana na mahitaji ya mazingira haya?

2.3 Je! Wadhan zoezi la upuliziaji wa vidudu linatekelezeka na ni afua sahihi kwa ya Halmashauri hii?

2.4 Unaweza eleza ni kwa namna gani jamii ilishirikishwa katika utekelezaji wa zoezi la upuliziaji viuadudu?

2.5 Je! Ni kwa namna gani viongozi wa jamii walishiriki katika zoezi la upuliziaji viuadudu?

2.6 Je! Zoezi la kusaka mazalia ya mbu (maji ambayo yana viluwiluwi au uwezo wa kuwa chanzo cha mbu kuzaliana) lilifanyika katika mitaa/vijiji vyote ndani ya Halmashsuri hii?

2.7 Je! Mazalia yaliyo gundulika ni yote yaliweza kupuliziwa na dawa ya viuadudu? Kama sivyo ni sababu zipi zilipelekea kushindwa kupulizia viuadudu kwenye mazalia yaliyo salia?

2.8 Je! Ufuatiliaji ulifanyika kutathmini manufaa ya upuliziaji?

2.9 Je! Unaweza elezea nini kuhusu hali ya upatikanaji wa rasilimali iliyopo?(Itategemea matokeo ya ukaguzi wa bidhaa).

1. **Sababu wezeshi kwa utekelezaji wa upuliziaji viuadudu**
   1. Ni sababu zipi zimekuwa chanzo cha mafanikio ya zoezi la upuliziaji kwa kiwango kilicho fikiwa?
   2. Kuna dalili za zoezi la upuliziaji kuwa endelevu?

3.3 Ni sababu zipi zimeweza kuchochea ushiriki wa wananchi katika zoezi la upuliziaji?

3.4 Je! Kuna uwezekano wa ushiriki wenu kuwa endelevu huko mbeleni?

1. **Sababu zuizi kwa utekelezaji wa upuliziaji viuadudu**

4.1 Nis sababu zipi zimepelekea ufanisi wa upuliziaji kuwa duni? Je! Ni mbinu zipi ziliwahi tumika kutatua changamoto hizo? Je! Zilikuwa na manufaa? Je! Kuna namna nyingnine ya kutatua changamoto hizo?

4.2. Je! Kuna uhitaji wa kufanya mabadiliko au kuboresha namna ya usimamizi au sheria (hapa kijijini/mtaani au kwa halmashauri) ili kuongeza ushiriki wa wanajamii katika zoezi hili la upuliziaji wa viuadudu?

**Document Review Template**

| **Assessment of Resource Availability** | | | |
| --- | --- | --- | --- |
| **Item type** | Number of items required | Number of items available | Sufficiency  (% of demand) |
| **Equipment** | | | |
| Sketch map |  |  |  |
| Gumboots |  |  | Sufficiency  (% of demand) |
| Overalls |  |  |  |
| Dippers |  |  | Sufficiency  (% of demand) |
| Sprayer |  |  |  |
| **Fund availability** | | | |
|  |  |  |  |
| **Trained Personnel(COPRS)** |  |  |  |
| **Biolarvicide** |  |  |  |

| **Biolarvicide Implementation** | |
| --- | --- |
| **Activity** | **Frequency/number** |
| Total number of wards in the councils |  |
| Number of Wards reached for advocacy on biolarviciding to Ward Development Committee members |  |
| Total number of villages in councils |  |
| Number of villages reached for community sensitization on biolarviciding (at least once) |  |
| Number of villages reached for breeding site identification |  |
| Total number of breeding sites identified from all villages |  |
| Number of breeding sites applied with biolarvicide: | |
| At first round |  |
| At second round |  |
| At third round |  |
| At fourth round |  |
| Larval surveillance |  |

**Questionnaire for Vector Control Officers**

1. **Participant’s particulars**

Council of work (ID Number) ………………..

Age…………………………………..

Gender……………………………….

Education level………………………

Profession……………………………

1. **Assessment of resources for biolarviciding implementation**
   1. Was there fund budgeted to finance biolarviciding activities in the council for the year 2018/2019?
      - 1. Yes
        2. No
   2. If yes, was the fund for biolarvicide activities disbursed timely?
2. Yes
3. No
   1. What is the main source of funds for biolarviciding implementation?
      - 1. Council own source
        2. Community contributions
        3. Donors
        4. Others (specify)………………………
   2. How can you grade the level of financial availability for biolarviciding activities?
      - 1. Caters for less than 50% of demand
        2. Caters for 50% to 70% of demand
        3. Caters for more than 70% of demand
   3. Was there training on biolarviciding conducted to council Vector Control Officers?
      - 1. Yes
        2. No
   4. Was there training on biolarviciding conducted to Community Own Resource Persons in the council?
4. Yes
5. No
   1. What is the availability of trained Community Own Resource Persons?
6. No CORPs to all Wards
7. Less than one per ward
8. At least one per ward
   1. What is the availability of transport for biolarviciding implementation?
9. Not available
10. Available
    1. What is the level of biolarvicide supply in your council?
11. Low, if it caters for less than 50% of demand
12. Moderate, if it caters for 50 to 70% of demand
13. High, if it caters for more than 70% of demand
    1. What is the availability of equipment for biolarviciding in council?

1. Low, if it caters for less than 50% of demand

2. Moderate, if it caters for 50 to 70% of demand

3. High, if it caters for more than 70% of demand

1. **Community participation in biolarviciding implementation**
   1. Which group in the community were involved during need assessment for biolarviciding implementation?
2. Representatives at Full Council Committee
3. Representative at Ward Development Committee
4. Members of Ward Health Committee
5. Members of Village Health Committee
6. Others (specify)…….………………….
7. No group were involved
   1. What is the community organization for biolarviciding activities?
8. There is specific assignment of task for different group of people who are well coordinated
9. There is specific assignment of task but poor coordination
10. No known specific assignment of task for different group of people
    1. In what way does the community contribute for biolarviciding activities?
11. Contribution of fund for material purchases
12. Volunteer for activities
13. Contribution of equipment
14. None
    1. In what ways does the community leaders engage in biolarviciding activities?
15. Assist in community sensitization
16. Assist in resource mobilisation
17. Assist in management
18. None.
